# Mechanisms of rumination change in adolescent depression (RuMeChange): study protocol for a randomised controlled trial of rumination-focused cognitive behavioural therapy to reduce ruminative habit and risk of depressive relapse in high-ruminating adolescents

**DOI:** 10.1186/s12888-021-03193-3

**Published:** 2021-04-23

**Authors:** Henrietta Roberts, Rachel H. Jacobs, Katie L. Bessette, Sheila E. Crowell, Mindy Westlund-Schreiner, Leah Thomas, Rebecca E. Easter, Stephanie L. Pocius, Alina Dillahunt, Summer Frandsen, Briana Schubert, Brian Farstead, Patricia Kerig, Robert C. Welsh, David Jago, Scott A. Langenecker, Edward R. Watkins

**Affiliations:** 1 Mood Disorders Centre, School of Psychology, Sir Henry Wellcome Building for Mood Disorders Research, University of Exeter, Exeter, EX4 4LN UK; 2 University of Chicago, Chicago, IL 60612 USA; 3 Department of Psychiatry, University of Utah, Salt Lake City, UT 84108 USA; 4 Department of Psychology, University of Utah, Salt Lake City, UT 84108 USA

**Keywords:** Rumination, Depression, Adolescence, Cognitive behavioral therapy, Development, Resting state networks

## Abstract

**Background:**

Adolescent-onset depression often results in a chronic and recurrent course, and is associated with worse outcomes relative to adult-onset depression. Targeting habitual depressive rumination, a specific known risk factor for relapse, may improve clinical outcomes for adolescents who have experienced a depressive episode. Randomized controlled trials (RCTs) thus far have demonstrated that rumination-focused cognitive behavioral therapy (RFCBT) reduces depressive symptoms and relapse rates in patients with residual depression and adolescents and young adults with elevated rumination. This was also observed in a pilot RCT of adolescents at risk for depressive relapse. Rumination can be measured at the self-report, behavioral, and neural levels- using patterns of connectivity between the Default Mode Network (DMN) and Cognitive Control Network (CCN). Disrupted connectivity is a putative important mechanism for understanding reduced rumination via RFCBT. A feasibility trial in adolescents found that reductions in connectivity between DMN and CCN regions following RFCBT were correlated with change in rumination and depressive symptoms.

**Method:**

This is a phase III two-arm, two-stage, RCT of depression prevention. The trial tests whether RFCBT reduces identified risk factors for depressive relapse (rumination, patterns of neural connectivity, and depressive symptoms) in adolescents with partially or fully remitted depression and elevated rumination. In the first stage, RFCBT is compared to treatment as usual within the community. In the second stage, the comparator condition is relaxation therapy. Primary outcomes will be (a) reductions in depressive rumination, assessed using the Rumination Response Scale, and (b) reductions in resting state functional magnetic resonance imaging connectivity of DMN (posterior cingulate cortex) to CCN (inferior frontal gyrus), at 16 weeks post-randomization. Secondary outcomes include change in symptoms of depression following treatment, recurrence of depression over 12 months post-intervention period, and whether engagement with therapy homework (as a dose measure) is related to changes in the primary outcomes.

**Discussion:**

RFCBT will be evaluated as a putative preventive therapy to reduce the risk of depressive relapse in adolescents, and influence the identified self-report, behavioral, and neural mechanisms of change. Understanding mechanisms that underlie change in rumination is necessary to improve and further disseminate preventive interventions.

**Trial registration:**

ClinicalTrials.gov Identifier: NCT03859297, registered 01 March 2019.

## Background

Depression is a leading cause of disability, affecting more than 264 million people worldwide [1, 2]. The prevalence of depression has increased by nearly 20% in the last decade. Notably, the greatest increase in incidence for depression is during adolescence and early adulthood. In addition, 25% of individuals experience their first depressive episode before the age of 19 [1–4]. Despite the availability of well-established treatment options (e.g., cognitive behavioral therapy, CBT; antidepressant medications), significant challenges in the prevention and treatment of depression remain. There is considerable evidence indicating that depression is sub-optimally treated by existing psychosocial and pharmaceutical interventions (e.g., [5–7]), with a 40-60% remission rate. Of those in remission, 30-50% experience residual symptoms at the end of treatment [8–10], with high rates of relapse. A recent meta-analysis of adults reported that 29% of patients experienced relapse within 1 year and 56% experienced a recurrence within 2 years [11], with similar statistics in adolescents [12], emphasizing that for many, depression has a chronic recurrent course.

Adolescence is a key developmental window in which to intervene with individuals who are vulnerable to depressive episodes [13], as depressive illness has cumulative educational, psychosocial, occupational, and economic burden. Moreover, adolescent-onset depression typically follows a chronic, recurrent course, and is associated with more negative outcomes relative to adult-onset depression [14–21]. Evidence also exists that the recurrent form of depression tends to worsen as individuals transition into adulthood, with more severe and longer episodes [22]. However, current gold-standard treatments do not successfully treat a substantial number of adolescents (30-60%) and are not effective in reducing risk of relapse [12]. As a consequence, there is a rapidly expanding literature investigating interventions to reduce depression in adolescence and emerging adulthood, and to prevent relapse and recurrence in this age group [23]. Finally, adolescence is a period in which healthy habit development might achieve sustained benefits.

One proposed means to reduce depression recurrence in young people is to use preventive interventions that target specific proximal risk factors that have an established role in the onset, maintenance, and recurrence of depression. Meta-analyses have reported that whereas universal preventive interventions (aimed at whole populations) achieve only small-to-non significant effect sizes [24], targeted interventions (aimed at individuals presenting with subclinical symptoms and/or established risk factors) have larger reductions in hazard ratios, and these effects are sustained over a longer period [25, 26]. Within the framework of these proximal risk factors, there is an increased appreciation that the mechanisms underlying increased risk for psychopathology may be dimensional in nature, and may even be transdiagnostic [27–29]. For example, the Research Domain Criteria (RDoC) framework by the National Institute of Mental Health (NIMH) proposes dimensional risks for illness can exist along a continuum and may not be specific to a given disorder [27, 28]. Novel interventions should therefore seek to alter such mechanisms in order to effectively target the emergence and maintenance of mental health difficulties such as depression. Building on this framework, prevention mechanism trials [30] seek to establish whether interventions can reduce specific risk factors for psychopathology or increase established resilience factors.

Rumination is a known proximal risk factor implicated in the onset, maintenance and relapse of depression, and has been identified as a malleable target for intervention [31, 32]. Prospective longitudinal research has demonstrated that degree of ruminative habit (i.e., the frequency, intensity and automaticity of ruminative thought in response to negative affect) predicts (a) onset and duration of major depressive episodes [33, 34]; (b) depressive symptoms, after controlling for baseline depression and anxiety, across a range of follow-up periods [35, 36]; and (c) slower response to CBT and antidepressant treatment, as well as reduced likelihood of recovery following treatment [37, 38]. Degree of ruminative habit also mediates the effects of other identified risk factors on depressive onset [39]. Importantly, rumination is a sensitive, relevant risk factor for adolescent depression and prospectively predicts fluctuations in depressive symptoms over time in this age group [33, 40, 41]. Developmental studies indicate that the ruminative response style emerges and stabilizes during the transition from childhood to adolescence, consolidating into an emotionally pernicious habit that is strongly predictive of psychopathology in adolescence (see [42] for recent review). The emergence of rumination coincides with a period of rapid cognitive and neural development, during which continued, significant structural and functional changes are observed within the cognitive control network (CCN) and increasing connectivity within the default mode network (DMN) [42–48]. It has been suggested that increasing coherence within these brain networks may be important to understanding the emergence of rumination as a habitual response style during this developmental window [42].

Rumination has been related to abnormal functioning of key nodes and networks of the brain. From resting state and mood induction studies, evidence is present that rumination is associated with elevated activation and connectivity between DMN nodes (e.g., [49]), increased connectivity between CCN and DMN, and reduced connectivity within CCN. The DMN is thought to support self-referential processing, passive waiting, and attention to the internal environment. Both resting state-functional MRI (rs-fMRI) and task-based approaches have been employed to study the neural correlates of rumination among adults, and to a lesser extent, adolescents with depression [45, 49–58]. The temporal correlation of blood-oxygen level dependent (BOLD) activation, and perhaps synchronization and engagement, of specified nodes of the brain are reflected in rs-fMRI measures. Self-reported rumination is related to activation in regions of the DMN during task-based studies, including the dorsomedial prefrontal cortex (dmPFC), posterior cingulate cortex (PCC), and also visual and somatosensory areas. Alternatively, activation in the superior frontal gyrus in the CCN has been found during rumination induction tasks [48, 50, 52]. The CCN is a distinct network of distributed neural nodes that broadly supports integrative executive functions such as inhibitory control, working memory, and sustained complex attention [59]. One function of inhibitory control is its ability to modulate negative affective processes, including rumination [46, 60, 61]. Consistent with a CCN disruption hypothesis of elevated rumination, rs-fMRI studies have reported that disrupted/elevated DMN-CCN connectivity (and decreased within CCN connectivity) is associated with increased rumination and decreased inhibitory control [46, 61, 62]. Moreover, behavioral disruption is evident in the context of induced rumination in the laboratory, with more errors and greater interference (slower response times) observed during a concurrent attentional task [48, 63]. As such, the maladaptive effects of rumination can be observed at both the clinical level, in elevated risk for depression, and at the neural and behavioral level, including impaired performance on cognitive tasks and altered functioning of key brain networks.

A recent systematic review examined whether treatments for depression that specifically target rumination produce better outcomes than more generic treatments that do not target rumination [64]. Results showed that treatments specifically targeting rumination had significantly larger effect sizes in reducing rumination than other treatments. These effects on rumination post-treatment were significantly associated with reductions in depression severity only in variants of cognitive-behavioral therapy specifically designed to target rumination such as rumination-focused CBT (RFCBT) [64]. RFCBT differentiates between functional and dysfunctional styles of repetitive thinking; the more adaptive thinking style is conceptualized as being concrete and specific, whereas the unhelpful thinking style is abstract and evaluative, consistent with the phenomenology of depressive rumination. RFCBT conceptualizes ruminative thinking as an unhelpful habitual response and focuses on the use of functional analysis of rumination. Thus, RFCBT combines behavioral activation strategies with novel techniques to foster a concrete, process-focused style of thinking. In support of this model, an RCT comparing treatment-as-usual (TAU, including antidepressant medication) versus TAU plus RFCBT in patients with medication-refractory residual depression found that the addition of RFCBT reduced depressive symptoms more than medication alone [65]. RFCBT was found to improve remission rates (62% vs. 21%) and reduce relapse rates (10% vs. 53%) at 6 months post-treatment, with changes in rumination scores found to be a significant mediator of these effects. A further independent trial found that RFCBT improved depressive symptoms and reduced rumination, relative to a waiting list control group, in patients with residual depression [66]. More recently, RFCBT has been evaluated in adolescents and young people and as a preventive treatment for youth at increased risk for depression with promising results. In a high-risk preventive design, group and internet RFCBT were compared to a waiting list control condition in a large sample of Dutch adolescents and young adults with elevated rumination, but without current depression. Relative to the waiting list control, both RFCBT interventions significantly reduced rumination and depression at post-intervention and one-year follow-up, and halved the one-year incidence rates of depression [67]. In another preventive study, guided online RFCBT significantly reduced the subsequent onset of major depression, relative to a usual practice control, in UK undergraduates with elevated rumination, especially for those young people reporting higher levels of stress at baseline [68].

Importantly, a recent pilot RCT compared 8 weeks of TAU+RFCBT to TAU+assessment in 33 adolescents with a history of major depression who were at risk of relapse and observed that RFCBT significantly reduced rumination and depression relative to the TAU control group [69]. fMRI scans pre- and post-intervention showed that adolescents who received RFCBT demonstrated significant decreases in rs-fMRI connectivity between regions of DMN (the left posterior cingulate cortex) and the CCN (the right inferior frontal gyrus and bilateral inferior temporal gyri). The degree of change in connectivity was correlated with changes in self-reported depression and rumination, suggesting that the DMN and CCN may begin to function more independently (or in less conflict) as rumination reduces. RFCBT therefore is a promising candidate intervention for the prevention of depressive relapse and recurrence in adolescents following prior depressive episode(s), and may improve longer term outcomes for this at-risk population.

These findings indicate that RFCBT is able to modify rumination in adolescents, and shows potential as a targeted intervention to reduce risk of depressive relapse in high-risk adolescents. However, no full-scale study has evaluated RFCBT adapted for adolescents at risk of depressive relapse. Moreover, research has not yet revealed the neural mechanisms underlying increases or decreases in rumination, either in the moment or over time. This knowledge regarding the neural processes affecting rumination is particularly pertinent during the critical developmental period of adolescence, when ruminative habits typically emerge.

## Aims and objectives

The primary aim of this phase III, two-stage prevention mechanism randomised controlled trial is to evaluate whether RFCBT is effective in reducing the primary outcomes of (a) self-reported rumination and (b) rs-fMRI connectivity between regions of the DMN and CCN, when compared to a TAU + assessment-only (AO) control arm in the first stage (NIMH R61 grant phase) (assessments performed within context of the study and TAU provided in community), assessed at primary endpoint of 16-20 weeks post-baseline. These indices are chosen as potential predictors of risk for future depression; that is, this operates as a targeted trial of a prevention mechanism [30, 70]. The second stage (potential NIMH R33 grant phase if Go targets are met in first stage) includes comparison of RFCBT to relaxation therapy on the same primary outcomes. Secondary outcomes include change in symptoms of depression at 16 weeks, recurrence of depression over 12 months post-intervention period, and whether engagement with therapy homework (as a dose measure) is related to changes in the primary outcomes.

## Methods

### Study design

The study consists of a two-arm parallel-group single-blind randomised controlled trial. Stage 1 compares RFCBT to TAU + AO; stage 2 compares RFCBT to TAU + relaxation. The assessment team members will be blind to treatment arm. Our primary hypotheses are that RFCBT will be superior to the control arm in reducing (a) rumination, and (b) rs-fMRI connectivity between DMN and CCN. Our secondary hypotheses are that (a) that RFCBT will be superior to the control arm in reducing recurrence of depression over 12 months; and (b) engagement with RFCBT homework (dose response) will be related to changes in (i) rumination, (ii) DMN and CCN resting state connectivity, and (iii) degree of change in regional brain activation during a rumination induction from pre- to post- treatment. The study design was published in the National Clinical Trials Website (NCT03859297) in March 2019. The only substantive change is the move to tele-assessment and teletherapy in March 2020 in response to the COVID-19 pandemic. The neuroimaging protocol was discontinued from March through June 2020, and then reinstated with COVID-19 safety precautions.

### Study setting

The study takes place at the University of Utah in the United States of America. Participants attend assessment appointments at the Huntsman Mental Health Institute (formerly University Neuropsychiatric Institute) on the University of Utah campus, which provides primary inpatient and outpatient psychiatric services to young people and adults who are primarily residents throughout the Salt Lake Valley and Intermountain West region.

### Eligibility criteria

Eligible participants will be postpubertal adolescents aged 14-17 with a previous diagnosis of Major Depressive Disorder (MDD), based on the Diagnostic and Statistical Manual (DSM-5) [71] criteria and confirmed by assessments using the Kiddie Schedule for Affective Disorders and Schizophrenia – Present and Lifetime Version DSM-5 (KSADS-PL) [72, 73] (*N* = 60 in stage 1 and *N* = 120 in stage 2). Participants must have been in full or partial remission for a minimum of 2 weeks (typically much longer); partial remission is defined as meeting less than five clinical-threshold symptoms for MDD according to the KSADS-PL. In addition, participants will be required to have elevated levels of rumination, defined as an elevated Rumination Response Scale (RRS) [35]. Age-adjusted RRS threshold cut-offs for males are from 28 to 31 and for females from 35 to 38; the minimum cut-off levels increase with participant age [35, 74].

Exclusion criteria include a lifetime history of conduct disorder, autism spectrum disorder, any psychotic disorder (or psychotic episode unexplained by other known medical causes), or bipolar disorder, an estimated intelligence quotient (IQ) of 75 or less as estimated by a computer adaptation of the Synonym Knowledge subtest [75]; elevated current depressive symptoms as indicated by a raw score greater than 45 on the Children’s Depression Rating Scale – Revised (CDRS-R) [76]; endorsing suicide attempt or plan within the past 6 months, as assessed by the KSADS-PL, CDRS-R, and Lifetime Suicide Attempt Self-Injury Interview (L-SASI) [77]; reporting anorexia/bulimia or alcohol/substance abuse within the past 6 months. Currently receiving psychotropic medication other than stimulants for ADHD and antidepressants (e.g., anxiolytics and most atypical antipsychotics) will be exclusionary with the exception of those that are prescribed for treating depression only. If taking medications, participants will be required to have been on a stable dose of medication for the past 4 weeks, with no change in specific medication for past 6 weeks. The study will also exclude individuals reporting current or recent (past year) treatment with CBT, or related variants (e.g., detailed-oriented structured therapy involving elements such as cognitive restructuring, homework, or a CBT focus), and those reporting prior treatment with rumination focus. Due to residual concerns from institutional review boards about the brain imaging protocol, individuals who are currently pregnant are excluded. For safety and data quality reasons, those who have MRI contraindications including non-removable metal braces, tattoos with metal, or claustrophobia are excluded. In instances where potential participants either decline consent or provide consent but do not meet eligibility requirements, we will offer a list of clinically relevant treatment resources outside of the study, including the option of receiving the same treatment in outpatient clinic from one of the investigators. We will keep information collected from participants who consented and were later deemed ineligible for the purposes of assessing generalizability and sample representativeness.

### Recruitment

Participants for the proposed study will be recruited from the University of Utah Hospitals and Clinics and from a number of additional sources and organisations (e.g., The Balanced Mind Foundation, National Alliance on Mental Illness, American Foundation for Suicide Prevention), via advertisements on local radio stations (e.g., KSL radio), in schools, on social media (e.g. Facebook, NextDoor), and on Utah public transit (between May 2019 and May 2021 (estimated)). Adolescent/young adult-based hospitals and clinics, as well as high schools and universities in the Salt Lake City area, will be locations targeted for additional recruitment. Our successful retention rates with pilot work (approximately 83% over 2 years [69]) will be bolstered through close contact with all study participants and close monitoring at weekly research team meetings. If obstacles to study recruitment and retention are encountered, contingency plans such as increased advertisement, increased resources to cover research assistant time focused on recruitment outreach efforts, announcements to colleagues, and scheduled calls to participants, will be developed to address these challenges in collaboration with the team. Following verification of initial eligibility via telephone screening, participants are scheduled for a baseline visit during which we obtain complete written informed parental consent and adolescent participant assent prior to enrolment in the study. Parents will also be asked to consent to contribute information about the adolescent and relevant family medical history. For adolescents who turn 18-years-old during the course of the study, they will be asked to provide written informed consent to continue in the study once they are aged 18. Further eligibility will be determined by the research team via administration of the KSADS-PL, CDRS-R, RRS, and Synonyms Knowledge Test at the baseline visit. After the final baseline assessment procedures (diagnostic, neuropsychological, fMRI, and the stress and relaxation session), the patients will be randomly assigned to receive RFCBT or participate in the control group.

### Participant timeline

**Fig. 1:**
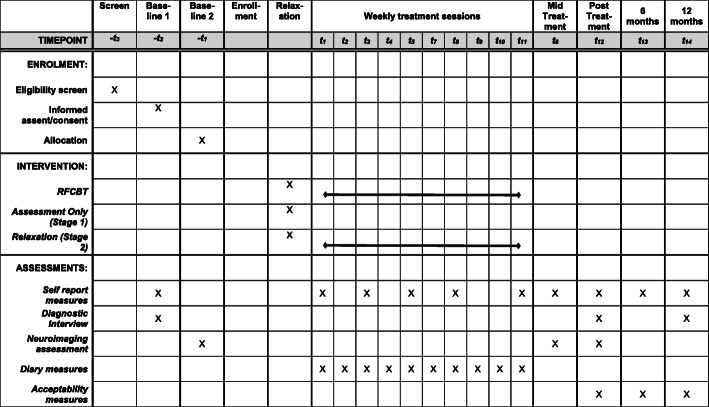
RuMeChange schedule of enrolment, interventions, and assessments

**Fig. 2:**
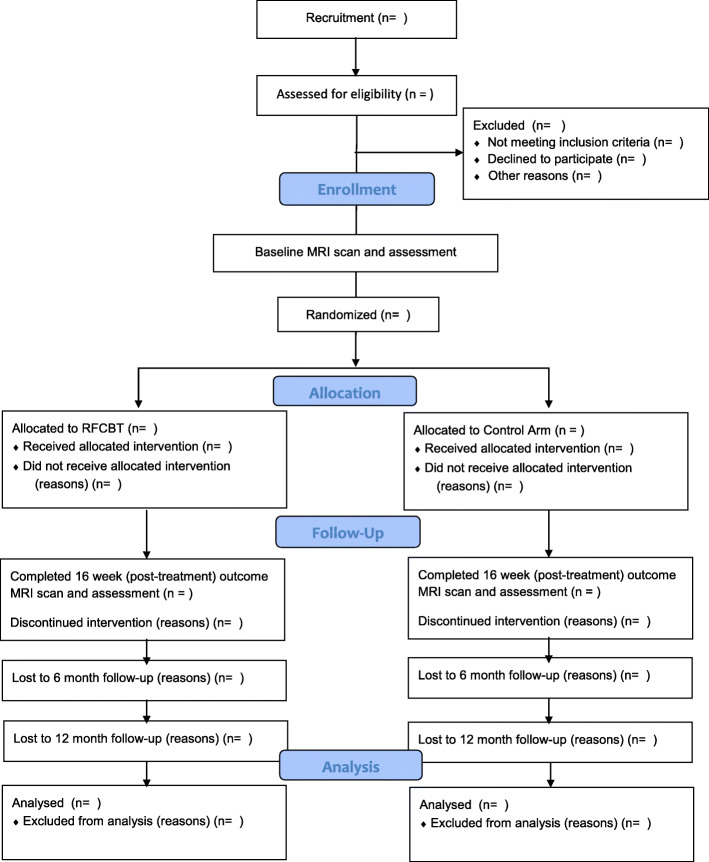
CONSORT flow diagram for RuMeChange

For further details on participant timeline, see Figs. 1 and 2.

### Sample size

This trial was funded as the first stage (R61 grant) in a potential two-stage study, with the second stage (R33 grant) comparing RFCBT to an active control of relaxation therapy. Moving to the second stage is contingent on the first stage meeting the Go criteria of baseline to post-intervention between-treatment arm effect size changes of .5SD (effect size) in RRS and .5SD (effect size) in rs-fMRI connectivity from left PCC to right IFG. These effect sizes were chosen to reflect a noticeable clinical difference, necessary to progress to second stage. For this stage, the target recruitment is *n* = 30 per group. Power was computed using gPower 3.1 for a repeated measurements model in the first stage (R61), to compare the effects of RFCBT versus TAU + AO where observations are nested within subjects, targeting a .5SD (effect) size change, and with known reliability of RRS (*r* = .77) and rs-fMRI connectivity of left PCC to right IFG (*r* = .71). On this basis, a target recruitment of *n* = 30 per treatment arm is estimated to provide power of .99 for RRS and .98 for rs-fMRI. Assuming up to 15% follow-up attrition at post-intervention follow-up, power remains >.94 for both comparisons. For the R33 stage, there will be an active treatment comparator so the effect size difference is likely to be smaller. To achieve adequate power to detect a meaningful clinical difference, we assumed a minimum difference of 0.22 SD for each comparison; for power at 0.80, alpha at 0.05, assuming 15% follow-up attrition, we need to recruit 60 young people per treatment arm (*N* = 120).

### Randomisation and allocation concealment

Block randomization will be performed using Matlab to produce computer-generated random codes that are stratified according to key clinical indicators and demographic variables, including RRS, CDRS, sex and age. Block randomization will be conducted by individuals (RWC, LRT) who will not share rumination scores, be providing therapy, or conducting independent assessments and will therefore be independent of the assessment and treatment staff. After each of four waves, the effect of randomization on age, sex distribution, RRS score, and proportion randomized to each arm are evaluated, and the formula is adjusted to maximize equivalence of the two groups.

The independent evaluators (IEs) who conduct diagnostics at baseline and follow-up visits will have separate calendars and channels of contact from the therapists and coordinators. Assignment of therapist will be based upon mutual alignment of schedules of the adolescent and therapist. When more than one therapist is available in an aligned slot, therapist caseload and adolescent preference (male or female therapist) will be taken into consideration. IEs who complete evaluations (for presence of mood disorder, mood symptoms) after the assessment period will remain blinded to treatment. We will report the extent to which this is successful, and the proportion of post-treatment evaluations that are conducted by blinded IEs.

IEs will administer the intake baseline assessment, the post-treatment assessment (16-20 weeks following baseline), as well as subsequent 12 month post-intervention follow-up interviews. Between the baseline and post-treatment assessment, adolescents will additionally report bi-weekly RRS measurements that are not shared with the therapist. To avoid accidental unblinding of IEs, a research assistant will meet with the participant and legal guardian prior to each diagnostic evaluation, in order to remind them of the importance of not disclosing this information to their assessor. Fidelity of blinding will be monitored with a blindness questionnairecompleted by IEs during follow-up assessments as noted above.

There is a detailed protocol to address suicidal ideation and risk, in which further questions are used to ascertain risk and appropriate actions are taken including consulting with supervisor, discussing with family, contacting crisis resources, or inpatient hospitalization. The trial also has a trained adolescent psychiatrist as independent study monitor to evaluate and address any safety issues.

### Interventions

Following a pilot study [69], remitted MDD adolescents will be randomised to AO as a comparison to RFCBT (Stage 1 of the trial). This is because RFCBT is not a common treatment in use in clinical settings, and has not previously been used in clinical trials of adolescents with remitted depression. This initial phase will therefore establish that RFCBT is effective in reducing rumination and changing the rs-fMRI connectivity of neural networks as predicted in this population. This comparison will additionally provide clinical decision metrics on who will benefit the most from RFCBT, which can be taken forward to refine the treatment protocol for Stage 2, in which RFCBT is compared to relaxation. All participants, regardless of arm, will receive one session of practice in progressive muscle relaxation, before progressing to RFCBT or AO.

Adolescents randomised to RFCBT will meet with a therapist on a typically weekly basis for 45-60 min following the manualized intervention for a target of 10 to 14 sessions [78], typically over a 3 month period. Rumination is characterized by abstract thinking about the causes, meanings, and implications of symptoms and difficulties, as exemplified by asking “Why me?” questions, with this abstract thinking implicated in the negative consequences of rumination. In contrast, encouraging more concrete thinking, focused on how events happen and the context and sequence of what did happen can help an individual move into a specific action and plan, keep an event in perspective, and reduce depression. RFCBT targets rumination and other maladaptive forms of emotion regulation such as suppression and avoidance and provides skills training in effectively coping with rumination. RFCBT specifically addresses rumination through psychoeducation, adopting a functional analytic approach to the learned habitual behaviour of rumination, and a focus on shifting processing style [79–81]. The adolescent is taught to notice triggers for rumination as well as the consequences of rumination and how to shift into practicing a more adaptive strategy such as an attention training exercise, behavioural activation, thinking in a concrete way, self-compassion, or active problem-solving [79–81]. RFCBT directly teaches adolescents to recognize rumination and to notice the influence this behavior has on their mood. The acronym ASK is used to teach adolescents that, rather than ruminating, which tends to take the form of passive, abstract, and critical forms of thinking about oneself, to be Active, Specific, and Kind. Through regular functional analyses in sessions, adolescents learn about their cycle of emotions and how the habit of rumination can make it harder to take action. A key skill taught is to change abstract and general thinking about difficulties and problems into a more specific, concrete and contextualized approach. Patients are taught to spot their abstract overgeneralized thoughts and “Why?” questions and to replace these with more helpful “How?” questions. Consistent with changing a habit, adolescents repeatedly practice using these different techniques in response to their warning signs for rumination (IF-THEN strategic plans) [82, 83].

Trial therapists will be psychologists and masters-level therapists who have completed a minimum of 5 days of training in RFCBT with EW and RHJ, (the treatment author and PI of the pilot adolescent trial), over 4 separate sessions, and will receive weekly supervision sessions from the treatment author and expert supervisors throughout the intervention delivery, including direct audio or video review of sessions. Therapists will use individual session templates to record and track therapy content and ensure adherence to the treatment protocol (including session checklists and overall therapy adherence module checklists). Digital recordings of therapy sessions will be used for the purposes of clinical supervision and monitoring. Twenty percent of sessions will be randomly reviewed using an adapted version of the Cognitive Therapy Rating Scale [84], modified for the specific principles and techniques of RFCBT, by an independent rater blind to treatment progress in order to monitor fidelity.

Participants will be provided with paper handouts and electronic tablet devices that are set-up with access to the therapy and homework materials. These (or the individuals’ cell phones) will additionally deliver personalised prompts to complete an electronic therapy diary (through the PACO Survey application), in order to monitor homework practice and track the relationships between practice of therapy techniques and levels of rumination in everyday life.

TAU may occur during the course of the trial with the participant’s primary care physician, mental health service provider, or psychiatrist, including a referral resource list that is provided to every participant for emergency and outpatient mental health services. TAU received will be assessed at the follow-up assessments. The primary treatments provided in this age range are expected to be supportive therapy and medication management (we expect roughly half will be on stable medication).

In the second (R33) stage of the trial, relaxation training will occur parallel to RFCBT to control for the effects of therapy structure, time, therapist contact, use of an active coping strategy for mood modulation, and attention. As such, sessions will occur weekly for 45-60 min and will include progressive muscle relaxation, simple breathing techniques, and guided imagery that focus on bodily and somatic relaxation. Adolescents randomized to relaxation will also receive exercises to do as homework in between sessions, parallel to the RFCBT group. This includes adaptions of relaxation formats for different situations that may not allow for a full execution (e.g., an airplane runway, in an office chair). Given that relaxation techniques are empirically supported for alleviating distress, anxiety, and depression among many different populations, we expect some effect of relaxation but it does not specifically target the mechanism of rumination.

### Outcomes

Outcomes will be assessed from baseline (pre-randomisation) to 16-20 weeks and 12 months post-intervention (see Fig. 1).

#### Primary outcome

The primary treatment outcome is self-reported reductions in rumination, as assessed using the brooding scale of the RRS [36] from baseline to 16-20 weeks post-baseline. Brooding is defined as “passive and judgmental pondering of one’s mood” ([85], p. 99), and is associated with maladaptive strategies and depression. We will use the Reliable Change Index (RCI) as an indication of change in brooding rumination scores [86]. RCI is a measure of how much change in a measurement can be ascribed as a meaningful movement at a statistical level, for the individual case, based upon the error inmeasurement, reliability of an instrument, and moderating for the effects of regression to the mean. In pilot work with adolescents, 57% of those treated with RFCBT obtained a change in total rumination scores at or above an RCI level, suggesting an adequate dose of targeted therapy (vs. 14% of AO group, e.g., random reduction).

The primary brain target (capturing the neural mechanisms compensating for rumination as a result of RFCBT) is elevated connectivity between the DMN and CCN (and diminished within-CCN connectivity) during rs-fMRI [46, 87]. Resting state fMRI (rs-fMRI) will investigate concurrent (or synchronized) changes in blood flow while adolescents are at rest with eyes open looking at a fixation cross. All adolescents will complete two identical MRI scans: at baseline and then at post-intervention (16 weeks), corresponding to the end of RFCBT sessions. A 64-channel coil will be used, and rs-fMRI sequences will be consistent with the Adolescent Brain Cognitive Development (ABCD) study protocol, with same TR, TE, and multiband sequences. We will collect 4 rs-fMRI runs at each of the two scans. Seed-based analyses will be used to probe changes to functional connectivity. The primary neural outcome is therefore reduced rs-fMRI connectivity between the left posterior cingulate cortex and right inferior frontal gyrus (nodes within the DMN and CCN respectively) [57]. Network-based analysis will also be probed for CCN-DMN changes in connectivity. Two experimental task-based paradigms [63, 88] will be employed to investigate directed induced rumination vs. distraction (block design), and uninstructed use of rumination during a sustained attention task [63]. These probes are explicitly designed to look at network function and dynamics of DMN nodes alone and with CCN, and how they may change selectively with RFCBT.

#### Secondary outcome

The occurrence and time to onset of any subsequent mood disorder and/or depressive episode are secondary outcomes. Recurrence of MDD and related mood disorders will be assessed by diagnostic interview (KSADS-PL) administered by IEs at post-intervention and at 12 months.

Dose is a rating of homework completion and skill integration (rated by the clinician), and degree of rumination change (defined by RCI). The primary clinical outcome is therefore change on the RRS score (as captured by the RCI) after treatment. We will additionally examine the stability of RRS change at 6 and 12 months post-intervention.

### Data collection and management

To promote participant retention, the study coordinators will keep in contact with the families via their preferred method (e.g., text messaging, emailing, and telephone calls). The participant payment schedule will be spread out across visits, so that adolescents will be compensated for each time they attend an assessment. Follow-up appointments will be scheduled in advance and at the families’ convenience, and participants will be contacted before appointments to remind them of the appointment and confirm the date/location. In the event of a missed visit, participants will be contacted through email and telephone. We will then wait until the next assessment/visit and attempt to schedule the participant. If the participant is unresponsive after several missed visits without a response, they will be called directly by co-PI Langenecker. They will be asked if they wish to discontinue or if no response, they will be considered lost to follow-up. To enhance interest and retention, additional strategies will be employed, including sending a birthday postcard (if agreed), provision of a printed 2d picture of the adolescent’s brain (after the intervention period, if agreed), and a 3D model of the adolescent’s brain (at 12 month follow-up, if agreed).

The MRI data will be stored in mirrored RAID (redundant array of independent disks) arrays, which are both firewall-protected and isolated from access outside the immediate local network. The RAID arrays will also be used to store other data from the participants, and will be backed up daily with digital tape. All non-image subject data will bestored in REDCap. REDCap is a meta-data driven software solution that facilitates secure collaborative data access, real time data validation and auditing [89], and is compliant with the Health Insurance Portability and Accountability Act (HIPAA) and General Data Protection Regulation (GDPR) best practices [90, 91].

All data will be collected, stored and handled in accordance with HIPAA [90]. The study protocol, documents, data management, and safety plans have been approved by the University of Utah Institutional Review Board (IRB_00113733). Approval for sharing and analysis of deidentified and coded data has been obtained by the University of Exeter Psychology Ethics Committee and data sharing plans have been subject to ethical review to ensure these are compliant with the European Union GDPR [91]. It will be made clear to participants how their data will be used and stored prior to obtaining consent to participate in the trial. All participants will be assigned a unique ID; coding all study data and images with an identifying number and referring to this number in all analyses will preserve confidentiality. The participants will not be identified in any reports of behavioural, clinical, or functional data.

Two independent clinicians with expertise in cognitive behavioural therapy and in working with adolescents, as well as an independent statistician at Utah, compose the internal Data Safety and Monitoring Board (DSMB), which will meet in person at least once per year for routine review of records (review of protocol, of a prototypic record, review of all adverse events, and review of all protocol deviations), and at least every 6 months by telephone/videoconference, if there are any actionable items that occur for enrolment or conflict of interest concerns, or participant safety review. The Independent Study Monitor (ISM) (XongRaio, M.D., adolescent psychiatrist), in concert with the DSMB, will set up a study charter before enrolment of the first participant that includes enrolment data, safety data, and data integrity. The DSMB will review de-identified study data for data quality and integrity, adherence to the protocol, participant safety, study conduct and progress twice per year. The ISM and DSMB will be blinded to participant group assignment. In the case of specific incidents (expected or unexpected adverse events), the ISM or DSMB may request from the study team information pertaining to group assignment for a given individual, or aggregate information on a group of individuals who experienced an adverse event. The ISM and DSMB retain autonomy to make determinations and recommendations in the case of adverse events. As the treatments have already been used with adolescents in a pilot study and in a larger European trial [66–68], such modifications are not anticipated. The ISM or DSMB will issue a monitoring report to the PI following each review/meeting, including any significant actions taken and any final recommendation(s) with regard to the study’s continuation. The ISM will be responsible for reviewing trial stopping/pausing decisions and enrolment recommendations. The ISM will review, within 24 h, any suicide-related assessments or concerns. In cases with re-emerging depression without increased suicidal ideation, the ISM will be consulted after two consecutive elevated measurements and will then decide how the participant shall proceed. The participant may be asked to pause participation, in which cases referrals will be made to suitable clinical services. At such a time as when the adolescent is stable and low risk, they may resume participation in the trial. There are no anticipated stopping rules for the entire study. The intervention is a low-risk intervention in those who are currently low in symptoms, and families can pursue treatment as usual during the trial.

### Statistical methods

Primary analyses will be conducted on intention-to-treat basis (as randomised) and missing data assumed to be missing at least at random (MAR), confirmed using missing data analysis. Unplanned missing data will be handled via multiple imputation (MI). Secondary variables will be used to improve the estimation of missing data. Sensitivity analysis, assuming a variety of MI models (Missing at Random; Missing Not at Random), will confirm the probable impact of missing data.

To compare the effects of RFCBT with the control arm, mixed-effects longitudinal models will be used. The first analysis tests change in RRS scores; the second analysis tests change in connectivity between the left posterior cingulate cortex and right inferior frontal gyrus. For each, the random subject intercept parameter will explain the variation in measurements of subjects at baseline, whereas the random slope parameter will explain variation over time. The models will also incorporate within-subject variation. The group by time interaction parameter will be used to differentiate the trend of RFCBT from that of the control arm on each outcome. Sustainability of improvement will be measured utilizing data from post-intervention to until 12 months post-intervention. To measure relation between degree of daily engagement in homework and degree of reduction in rumination, we will calculate Pearson correlations. A contrast using pre and post measures of rs-fMRI between left PCC and right IFG in adolescents on the RFCBT arm will be compared to the control arm, as described in Jacobs et al. 2016 [57]. The same approach will be adopted for exploratory, task-based fMRI, capturing state rumination.

Preliminary data and published studies include targeted seed-based analyses of connectivity between PCC (DMN node) and left inferior temporal gyrus (CCN node) [46, 47, 58, 60, 62, 64–69]. Connectivity of PCC to inferior frontal gyrus (also CCN) was considered. Because rs-fMRI analyses are rapidly evolving, there may be additional analytic strategies that evolve between now and interim study analysis – we will co-publish any use of these new techniques in the same paper with the primary seed-based analysis. We will evaluate and publish a Bayesian technique to allow for subtle variations in seed and node 3D coordinates. This is based upon observed sample to sample fluctuations in spatial smoothing, functional connectomic parcels, such that exact coordinates may not align for seed and node from sample to sample. As an example, our prior work shows similar, and only partially overlapping nodes for identification of PCC to superior frontal gyrus increased connectivity in MDD compared to healthy controls in three independently published samples [46].

Subsequent analyses will use the Complier Average Causal Effect (CACE) analysis [92, 93]. CACE assumes that the effect of treatment depends on the adherence to treatment (dose effects), which in turn is dependent on randomisation, and that randomisation has no direct effect on the outcome variable. As such, CACE takes into account adherence and compliance with the treatment in order to estimate a treatment effect, whilst also retaining the benefits of randomisation. Briefly, CACE estimates the difference in the outcome variable between the compliers in the treatment arm and the compliers in the control arm had they been offered the treatment, assuming that there are similar rates of compliance in both arms as a result of randomisation. Compliance will be defined as attending at least 8 out of a possible maximum of 16 RFCBT sessions.

## Discussion

Adolescent-onset depression often follows a chronic, recurrent course, and is associated with a number of long-term negative outcomes [14–21]. Understanding specific treatment mechanisms that can reduce short- and long-term risk for depressive relapse in young people who have experienced a depressive episode is therefore important in order to improve clinical outcomes. RFCBT was developed to specifically target maladaptive rumination, and has been found to be effective in reducing rumination and, subsequently,risk of depressive relapse in clinical trials with adults, and in a recent pilot trial with adolescents. Rumination can be measured at the neural level, in increased within-network DMN connectivity, and patterns of connectivity between DMN and CCN. The current study will use RFBCT in adolescents at risk of depressive relapse, with the goal of achieving both significant clinical change in rumination, and associated changes in connectivity between key DMN and CCN nodes that have been linked to decline in problematic rumination. Enhanced understanding of the neurocognitive mechanisms underpinning rumination and the prevention of depressive relapse could help to develop better targeted personalised intervention strategies.

## Data Availability

The study is registered on clinicaltrials.gov (NCT03859297), which will be updated with the published protocol and the study results and associated publications. De-identified data and results will be submitted to the National Database for Clinical Trials Related to Mental Illness (NDCT). NDCT provides a secure system to support the submission, sharing and access of relevant data at all levels of biological and behavioral organization and for all data types. Access to data for research purposes will be provided through the Data Access Committee. In accordance with the trial resource sharing agreement, data will additionally be deposited into the National Database for Autism Research, which is a US national database that houses deidentified data from many federal studies, irrespective of the disease/disorder/condition under study.

## Abbreviations

AO: Assessment Only
CACE: Complier Average Causal Effect
CDSR: Children’s Depression Rating Scale
CBT: Cognitive Behaviour Therapy
CCN: Cognitive Control Network
DSMB: Data Safety and Monitoring Board
DMN: Default Mode Network
DSM-V: Diagnostic and Statistical Manual fifth edition
dmPFC: dorsomedial prefrontal cortex
fMRI: functional magnetic resonance imaging
GDPR: General Data Protection Regulation
HIPAA: Health Insurance Portability and Accountability Act
ISM: Independent Study Monitor
KSADS-PL: Kiddie Schedule for Affective Disorders – Present and Lifetime Version
MDD: Major Depressive Disorder
MAR: Missing at least random
MI: Multiple imputation
NDCT: National Database for Clinical Trials
RCI: Reliable Change Index
rs-fMRI: resting-state functional magnetic resonance imaging
RFCBT: Rumination Focused Cognitive Behavioural Therapy
RRS: Rumination Response Scale
TAU: Treatment-as-usual
